# 1-(5-Bromo-2-hydroxy-4-methoxyphenyl)ethanone [SE1] Inhibits MMP-9 Expression by Regulating NF-κB and MAPKs Signaling Pathways in HT1080 Human Fibrosarcoma Cells

**DOI:** 10.1155/2018/5639486

**Published:** 2018-11-05

**Authors:** Fang Gong, YuanYuan Zhang, JiaChao Lin, ChengYong Li, ChunXia Zhou, PengZhi Hong, BoMi Ryu, Zhong-Ji Qian

**Affiliations:** ^1^College of Food Science and Technology, Guangdong Ocean University, Zhanjiang 524088, China; ^2^College of Chemistry and Environment, Guangdong Ocean University, Zhanjiang 524088, China; ^3^Shenzhen Institute of Guangdong Ocean University, Shenzhen 518114, China; ^4^Department of Marine Life Sciences, Jeju National University, Jeju 63243, Republic of Korea

## Abstract

Hippocampus is a traditional medicine in China, which can be used for treating tumors, aging, fatigue, thrombosis, inflammation, hypertension, prostatic hyperplasia, and other diseases. 1-(5-Bromo-2-hydroxy-4-methoxyphenyl)ethanone [SE1] from seahorse (*Hippocampus kud*a Bleeler) has been shown to suppress proinflammatory responses. In the present study, SE1 potently inhibited gelatin digestion by MMP-9 induced by phorbol 12-myristate 13-acetate (PMA) and migration of human fibrosarcoma HT1080 cells in dose-dependent manner. Moreover, western blot analysis and immunofluorescence analysis have been studied on MAPKs (ERK1/2, p38 kinase and JNK) and NF-*κ*B (p65 and I*κ*B), which refer to the clear molecular mechanism. The results indicated that SE1 significantly suppressed the phosphorylation of mitogen-activated protein kinases (MAPK: p38 kinase and JNK) and NF-*κ*B. Finally, molecular docking result showed SE1 interacts with TYR245 and HIS226 of MMP-9 by hydrogen bond and Pi-Pi bond to suppress MMP-9 activity. This data suggested that the SE1 may possess therapeutic and preventive potential for the treatment of MMP-9 related disorders.

## 1. Introduction

Tumor is the abnormal growth of tissue and the formation of mass [1]. The rapid proliferation and metastasis of tumor cells are a chief culprit of death of patients. Malignant tumor cells can invade into surrounding extracellular matrix (ECM) and degrade ECM with proteolytic enzymes to promote metastatic spread, which affect the body's normal function [2, 3]. Previous studies indicated that matrix metalloproteinases (MMPs) have the ability to degrade ECM and their overexpressions are to blame for tumor progression, invasion, metastasis, and angiogenesis [4–6]. Particularly, accumulating literatures have confirmed that MMP-2 and MMP-9 function as dominating enzymes taking part in degradation of type IV collagen which is a key component of ECM [7]. Among them, MMP-9 (92 kDa type IV collagenase, gelatinase B) is produced from human macrophages and polymorphonuclear leukocytes and associated with numerous pathological processes including cancer, inflammation, placental malaria, and cardiovascular diseases [8–11]. In addition, it has been well documented that both mitogen-activated protein kinases (MAPKs) and nuclear factor kappa (NF-*κ*B) signal pathways are related to tumor metastasis because they can control tumor cell migration and regulate MMPs expression [12, 13].

MAPKs connect extracellular stimuli to specific transcription factors and convert these signals into cellular responses in cell proliferation, differentiation, and apoptosis [14, 15]. MAPKs contain extracellular signal regulated kinase (ERK), c-Jun N-terminal kinase (JNK), and p38, and their activations are correlated with MMP-2 and MMP-9 expressions [16]. NF-*κ*B, a nuclear transcription factor, can regulate the expression of cytokines and adhesion factors which are important for cell differentiation, growth, adhesion, and apoptosis [17]. Furthermore, NF-*κ*B mediates the expressions of numerous genes that are related to tumor promotion, angiogenesis, metastasis, and MMPs expression [18, 19].

Seahorse, Hippocampus kuda Bleeler, a traditional medicine in China, can be used for treating tumors, aging, fatigue, thrombosis, inflammation, wheezing, nocturnal enuresis, hypertension, prostatic hyperplasia, and other diseases [20, 21]. SE1 is a major component in moutan cortex and a useful herb to treat atherosclerosis and infections. Himaya [22] indicated that SE1 could suppress proinflammatory responses. However, the antimetastatic capacity study of SE1 on HT1080 fibrosarcoma cells has not been discussed.

This study has focused on identification and investigation of MMP-9 inhibitory effects of SE1 with a clear understanding of underlying molecular mechanisms through MAPK and NF-*κ*B signaling pathway.

## 2. Materials and Methods

### 2.1. Materials and Reagents

**Figure 1:**
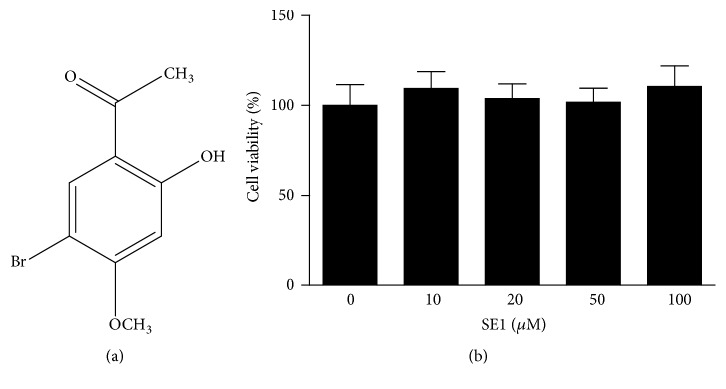
(a) Chemical structure of the 1-(5-bromo-2-hydroxy-4-methoxyphenyl)ethanone [SE1]. (b) Effect of SE1 on the viability of HT1080 cells. Cells grown in serum-free medium were treated with different concentrations of SE1 (10, 20, 50, and 100* μ*M) for 24 h and relative cell viability was assessed by the MTT assay. The results presented are the means ± S.D. of a triplicate experiment.

On the basis of the comprehensive spectral analysis and data published previously [22], SE1 was elucidated as a known 1-(5-bromo-2-hydroxy-4-methoxyphenyl)ethanone [SE1] (Figure 1(a)).

HT1080 cells were obtained from the Cell Bank of Chinese Academy of Sciences (Shanghai, China). Dulbecco's modified Eagle's minimal essential medium (DMEM), penicillin/streptomycin, and fetal bovine serum (FBS) were purchased from Gibco (Grand Island, NY). Gelatin A, Triton X-100, 3-(4,5-dimethylthiazol-2-yl)-2,5-diphenyltetrazolium bromide (MTT), and phorbol-12-myristate-13-acetate (PMA) were obtained from Sigma (St. Louis, MO, USA). Antibodies against MMP-9 (sc-6840), phospho-ERK1/2 (sc-81492), ERK1 (sc-94), phospho-p38 (sc-166182), p38 (sc-535), phospho-JNK (sc-6254), JNK (sc-345), phospho-NF*κ*B65 (sc-136548), NF*κ*Bp65 (sc-8008), phospho-I*κ*B*α* (sc-8404), I*κ*B*α* (sc-1643), and *β*-actin goat anti-rabbit IgG were obtained from Santa Cruz Biotechnology (Santa Cruz, CA, USA). Horse anti-mouse IgG were from Cell Signaling Technology (Beverly, MA, USA). The stock solution of 100 mM SE1 was prepared in dimethyl sulfoxide (DMSO) and stored at -20°C. Other reagents used in this study were of analytic grade.

### 2.2. Cell Culture and MTT Assay

HT1080 cells were cultured in DMEM containing 10% FBS and penicillin/streptomycin at 37°C in a humidified 5% CO_2_ incubator. To determine the cytotoxicity of the SE1, cells were seeded into 96-well plates at a density of 1×10^4^ cells/well and incubated with various concentrations of SE1 (10, 20, 50, and 100 *μ*M). At 24 h, 100 *μ*L MTT (1 mg mL^−1^) was added into each well for 4 h. Then, 100* μ*L DMSO was added to dissolve formazan crystals and the absorbance was measured at 540 nm.

### 2.3. Cell Migration

HT1080 cells were seeded in 24-well plate with various concentrations of SE1 (10, 20, 50, and 100* μ*M). The cell monolayer was scratched using a sterile pipette tip and then washed with PBS to remove cell debris. Cell migration across the wound was observed using a microscope (JiDi, GD30 China) and recorded photographically at 0, 12, and 24 h.

### 2.4. Determination of MMP-2/MMP-9 Activities

Activities of MMP-2 and MMP-9 in HT1080 cells were determined by gelatin zymography. Briefly, cells were seeded in 24-well plates with a density of 2×10^5^ cells/well and pretreated with different concentrations of SE1 (10, 20, 50, and 100* μ*M) for 1 h and stimulated by PMA (10 ng mL^−1^) for 72 h. Cell conditioned medium was collected to conduct gel electrophoresis, as described previously [23]. Finally, areas of gelatin hydrolyzed by MMPs were visualized as clear zones against blue background by Coomassie Blue staining and the intensities of the bands were estimated by ImageJ software (National Institute of Mental Health, Bethesda, Maryland, USA).

### 2.5. Western Blotting Analysis

HT1080 cells were incubated with SE1 (10, 20, 50, and 100* μ*M) for 1 h and then incubated with PMA (10 ng mL^−1^) for 24 h. Cells were collected and lysed in RIPA buffer. Equivalent amounts of proteins (20-40* μ*g) were separated by SDS-PAGE, as described previously [24], and subsequently transferred to NC membranes. Membrane was blocked with 5% skim milk at room temperature for 2 h and then incubated with primary antibodies. After being washed with TBST, the membranes were incubated with secondary antibodies for 2 h at room temperature and visualized with an enhanced chemiluminescence (ECL) detection system (Syngene, Cambridge, UK).

### 2.6. Immunocytochemistry for NF-*κ*B p65 Nuclear Translocation

Cells grown in 24 well plates were treated with SE1 for 1 h and then treated with PMA (10 ng/mL) for 24 h. After being washed with cold PBS three times, the cells were fixed with 4% paraformaldehyde (PFA). Then, cells were rinsed with PBS and permeabilized with 0.2% Triton X-100 for 10 min. The cells were blocked with 5% BSA in PBS for 1 h at room temperature. Localization of NF-*κ*B p65 was detected with anti-p65 antibody overnight at 4°C and incubated with secondary antibody for 2 h. After nuclear counterstaining with DAPI, images were taken using an inverted fluorescence microscope (Olympus Opticals, Tokyo, Japan).

### 2.7. Molecular Docking Analysis

The three-dimensional structures of MMP-9 (PDB: 4HMA) were downloaded by PDB. The structure of SE1 was built by using ChemDraw software. The protein and ligand molecules were prepared by Discovery Studio 3.5 software. Molecular docking of the SE1 into the MMP-9 protein binding site was performed by using CDOCKER protocol of DS 3.5. The small molecule conformation was searched by high temperature dynamics method, and they were optimized in the active sites area of the acceptor by simulated annealing.

### 2.8. Statistical Analysis

The data were presented as mean ± SD (n = 3). Statistical analyses were analyzed using t tests in GraphPad Prism 5 (GraphPad Software, San Diego, CA, USA). A value of P < 0.05 was considered statistically significant.

## 3. Results

### 3.1. Effect of SE1 on the HT1080 Cell Viability

The effect of SE1 on cell viability was estimated by MTT assay at four concentrations (10, 20, 50, and 100* μ*M). As shown in Figure 1(b), SE1 did not show a significant (P < 0.05) cytotoxicity of the cells on four concentrations. Therefore, the dose of 10, 20, 50, and 100* μ*M of SE1 was used for further experiments.

### 3.2. Effect of SE1 on Migration of HT1080 Cell

**Figure 2:**
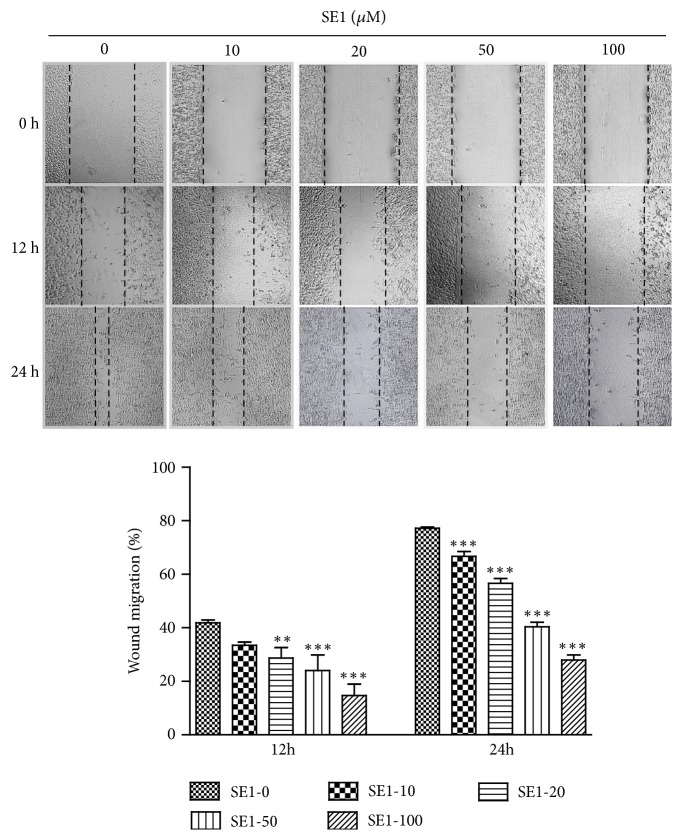
After scratch wounds were made on the confluent cell monolayer, the effects of SE1 on wound migration were monitored for 12 h and 24 h. Wound migration was measured in five selected fields and calculated based on the width of injury at 0 h. The results presented are the means ± S.D. of a triplicate experiment. *∗∗P* < 0.01 and *∗∗∗P* < 0.001.

In order to explore the effect of SE1 on migration of HT1080 cells, the migration assay was conducted. As shown in Figure 2, SE1 reduced the metastatic potential of H1080 cells in a dose- and time-dependent manner. Compared with untreated cells, the SE1 significantly (*P* < 0.05) reduced the ability of the cells to move around the wound after 12 and 24 h.

### 3.3. The Effect of SE1 on Expression and Proteolytic Activities of MMPs in HT1080 Cells

**Figure 3:**
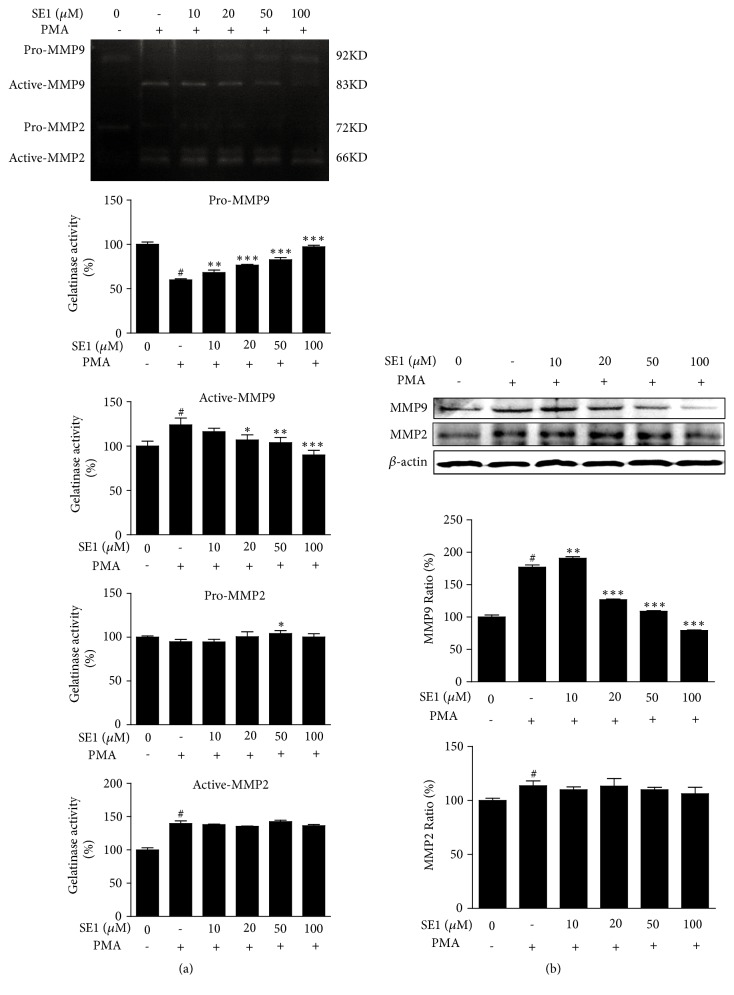
Effects of SE1 on activation and expression of MMP-9 and MMP-2 in HT1080 cells. (a) Gelatin zymography for the determination of MMP-2 and MMP-9 activities in SE1-treated HT1080 cells. HT1080 cells treated with SE1 (10, 20, 50, and 100* μ*M) for 1 h and stimulated by PMA (10 ng mL^−1^) for 72 h. Gelatinolytic activities of MMP-2 and MMP-9 in conditioned media were detected by electrophoresis of soluble protein on a gelatin containing 10% polyacrylamide gel. Untreated control was used as a loading control. (b) Expression of MMP-2 and MMP-9 in cell lysates was detected using western blot analysis. *β*-actin was used as a loading control. HT1080 cells treated with SE1 (10, 20, 50, and 100* μ*M) for 1 h and stimulated by PMA (10 ng mL^−1^) for 24 h. The relative amount of MMP-2 and MMP-9 was quantified by densitometry measurement (ImageJ). The results presented are the means ± S.D. of a triplicate experiment. ^*#*^*P* < 0.001, *∗P* < 0.05, ∗∗*P* < 0.01, and ∗∗∗*P* < 0.001, PMA stimulation.

The effects of SE1 on gelatinase activities and expressions of MMP-2 and MMP-9 were studied by gelatin zymography and western blot analysis. As depicted in Figure 3, compared with untreated cells, the exposure of PMA increased the activities and expressions of MMP-2 and MMP-9. However, the activities and expression of MMP-9 were reduced with SE1 treatment in a dose-dependent manner, and the inhibitory effect of SE1 on MMP-9 was more remarkable than MMP-2. The result revealed that the SE1 sufficiently suppresses the activities and expression of MMP-9.

### 3.4. The Effect of SE1 on p38, JNK Phosphorylation, and NF-*κ*B Activation in PMA-Induced HT1080 Cells

**Figure 4:**
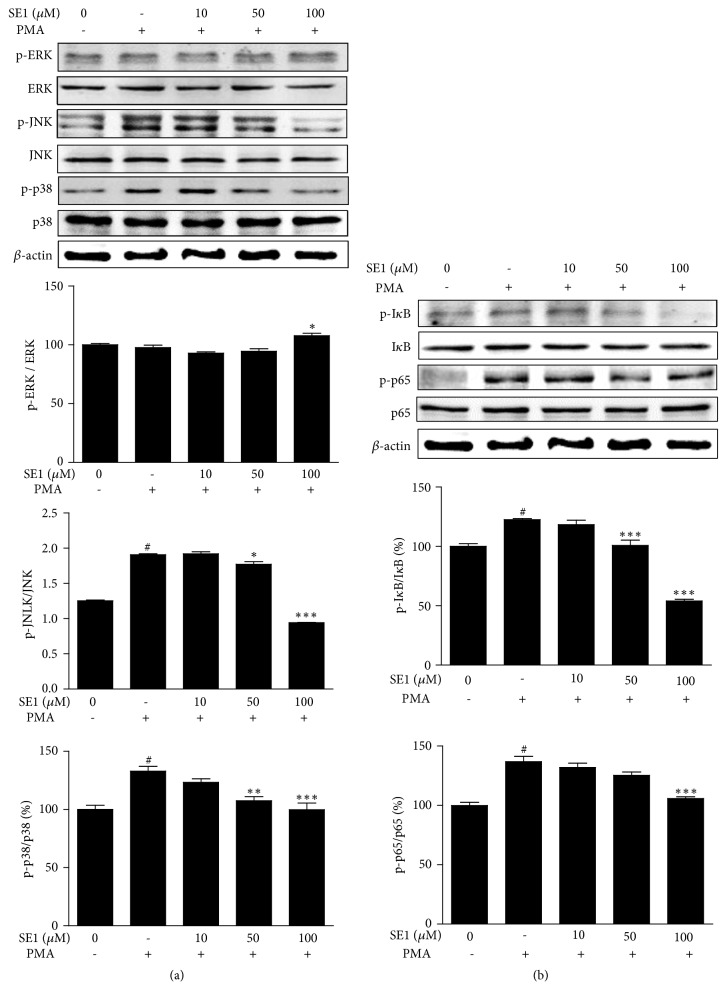
SE1 suppressed PMA-induced JNK, and NF-*κ*B activation in HT1080 cell. (a) Cells were pretreated with different concentrations (10, 50, and 100* μ*M) of SE1 for 1 h and PMA (10 ng mL^−1^) incubation was continued for 24 h. Total cell lysates were evaluated for MAPK phosphorylation/degradation using western blotting. Band intensities were normalized to *β*-actin expression, and then the relative ratios of phosphorylated form/total form were calculated. (b) The effect of SE1 on the nuclear translocation of the NF-*κ*Bp65 subunit under PMA stimulation was examined. *β*-actin was used as loading controls. The results presented are the means ± S.D. of a triplicate experiment. ^*#*^*P* < 0.001, *∗P* < 0.05, ∗∗*P* < 0.01, and ∗∗∗*P* < 0.001, PMA stimulation.

**Figure 5:**
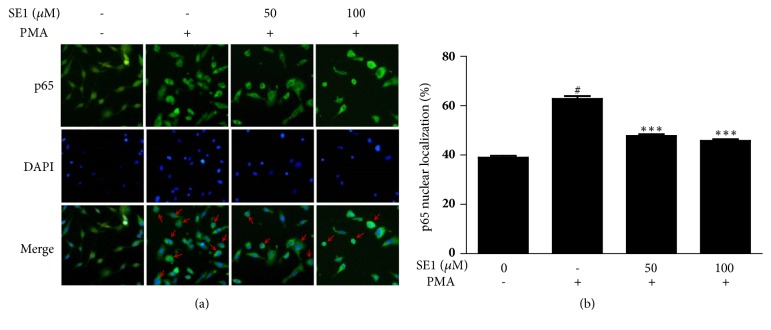
Cells were treated with 50 and 100* μ*M SE1 for 1 h and then stimulated with PMA (10 ng mL^−1^) for 24 h. (a) Nuclear translocation of NF-*κ*Bp65 was monitored by an overlay of blue DAPI staining with green p65 immunofluorescence. (b) p-65 nuclear localization was measured. Untreated control was used as a loading control. The results presented are the means ± S.D. of a triplicate experiment. ^#^*P* < 0.001 and ^∗∗∗^*P* < 0.001, PMA stimulation.

MAPKs and NF-*κ*B activation play crucial parts in the MMPs activity. Therefore, we further investigated whether SE1 has the ability to inhibit those two signaling pathways. Western blot analysis showed that PMA stimulation dramatically increased the levels of phosphorylation of p38 kinase and JNK. The treatment of SE1 dose-dependently decreased phosphorylation of p38 and JNK (Figure 4). Furthermore, I*κ*B*α* and NF*κ*B p65 phosphorylation were clearly increased by PMA induction, whereas, in SE1-treated cells, PMA-induced phosphorylation of I*κ*B*α* and NF-*κ*B p65 nuclear transfer was expressively blocked, as demonstrated by western blot (Figure 4) and immunocytochemistry (Figure 5). According to our data, SE1 reduced the expressions of MMP-9 in HT1080 cells through MAPK (JNK and p38 kinase) and NF-*κ*B pathways.

### 3.5. Molecular Docking Analysis

**Figure 6:**
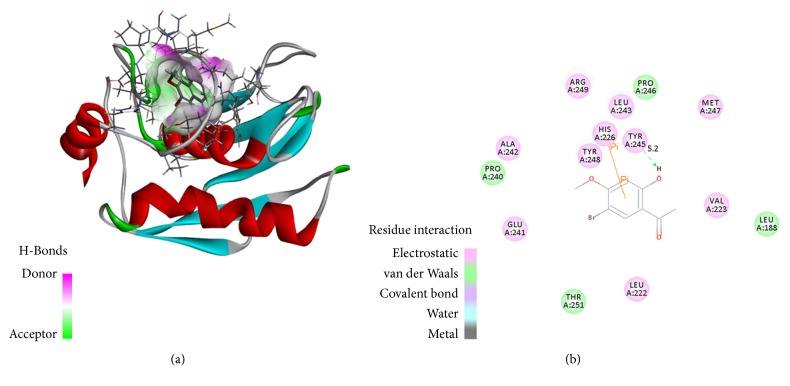
Interaction of the SE1 with MMP-9 active site. (a) Three-dimensional representation of ligand-4HMA interaction. (b) Two-dimensional representation of ligand-4HMA interaction.

The molecular docking was demonstrated to investigate the binding of MMP-9 and SE1, and experimentally verified contact residues of MMP-9 binding sites were selected as active site residues for molecular docking studies (Figure 6). At the end of docking, docked pose of MMP-9 with SE1 had CDOCKER energy of -28.0573 kcal/mol and CDOCKER interaction energy of -36.7308 kcal/mol. Amino acid residues TYR245 generated hydrogen bonding interaction and HIS226, Pi-Pi stacking interaction.

## 4. Discussion

In this study, we demonstrated that SE1 did not show a significant (P < 0.05) cytotoxicity on HT1080 cells of all tested concentrations (10, 20, 50, and 100* μ*M) (Figure 1(b)). And then we found that SE1 markedly decreased cell migration (Figure 2) without cytotoxicity. Cell migration is an important step in course of tumor development.

The tumor cells can proliferate continually. And then they detach from primary tumor and invade circumambient tissues and blood. If they are not killed by lymphatic cells, the tumor cell would migrate in vessels of the blood stream. Finally, these cells proliferate and produce a new tumor tissue [25, 26]. The degradation of EMC is important for the metastasis of tumor cells. In particular, MMPs could degrade the surrounding matrix and promote the metastasis of tumor cells, so they play a crucial role in the metastasis of tumor cells [27]. In the MMPs, both MMP-9 and MMP-2 are pivotal protease because they can degrade type IV collagen, which is the main component of the basement membrane [28]. In our study, SE1 treatment suppresses MMP-9 activities in PMA-induced HT1080 cells measured by gelatin zymography assay (Figure 3(a)), and then SE1 effectively inhibits MMP-9 expression in western blotting analysis (Figure 3(b)).

Previous study has found that the cellular migration of various cancer cell types was regulated by MAPKs signaling pathway, including ERK1/2, JNK, and p-38 [29]. Zhang [30] indicated that Ewing's sarcoma cell invasiveness and metastasis were inhibited by targeting p38 and JNK. The p38 MAPK signaling pathway plays an importance role in downregulating MMP-2 and MPP-9 expression [31]. Hepatocellular carcinoma metastasis was inhibited by suppressing the p38 and JNK/c-Jun signaling pathways [32]. Moreover, NF-*κ*B signal pathway seemed to be able to suppress tumor cell migration by blocking MMP-2 and MMP-9 expression [33–36]. Kim's and Cho's studies have shown, respectively, that suppression of p38, JNK, and NF-*κ*B activation could significantly inhibit cellular migration, invasion, proteolysis, and MMP-9 activity [37, 38]. To investigate whether SE1 inhibits the expression of MMPs and cell migration through MAPKs and NF-*κ*B pathways, we studied the phosphorylation of p38, JNK, and ERK of MAPKs and p65 and I*κ*B of NF-*κ*B, respectively, in PMA-stimulated HT1080 cells. Corresponding to former studies, our results showed that SE1 could inhibit the phosphorylation of p38 kinase and JNK (Figure 4(a)) and the phosphorylation of p65 and I*κ*B (Figure 4(b)). In summary, our results showed that SE1 reduces the metastatic ability of tumor cells by blocking expression and activities of MMP-9 and this was related to the downregulation of the NF-*κ*B and p38 and JNK signaling pathways.

Natural compounds and their derivatives have been shown as candidates for the treatment of tumor and can be the sources of therapeutic agents [39]. The present study showed that SE1 from seahorse obviously inhibited tumor metastasis and MMP-9 expression by PMA induced in HT1080 cells. It has been well documented that halogenated compounds can inhibit proteins that are involved in carcinogenesis [40] and halogens often provide high substrate specificity and contribute significantly to molecular recognition for the target enzymes and binding [41]. The presence of Br molecule in SE1 may contribute to its antitumor effect. And its small molecules size can cross the cell membrane and affect the expression and function of intracellular targets. In addition, the reaction of SE1 and amino acid residues TYR245 and HIS226 of MMP-9 inhibits the activity of MMP-9. The effect of SE1 on tumor cells and molecular docking indicated that SE1 is a good lead compound for tumor treatment.

## 5. Conclusion

In conclusion, SE1 isolated from seahorse was found to have the ability to reduce metastasis potential of HT1080 cells. In addition, SE1 markedly inhibits MMP-9 expression through blocking MAPKs (JNK and p-38 kinase) and NF-*κ*B signal pathways. In addition, molecular docking result showed that SE1 could interact with essential residues of MMP-9 (TYR245 and HIS226) by hydrogen-bonding and Pi-Pi stacking. Therefore, these results suggest that SE1 is a bioactive agent with antitumor metastasis effect, which might be a novel therapeutic candidate for treatment of tumor-related diseases.

## Data Availability

The data used to support the findings of this study are available from the corresponding author upon request.
